# Whole-exome Sequencing Analysis Identifies Mutations in the *EYS* Gene in Retinitis Pigmentosa in the Indian Population

**DOI:** 10.1038/srep19432

**Published:** 2016-01-20

**Authors:** Yanan Di, Lulin Huang, Periasamy Sundaresan, Shujin Li, Ramasamy Kim, Bibhuti Ballav Saikia, Chao Qu, Xiong Zhu, Yu Zhou, Zhilin Jiang, Lin Zhang, Ying Lin, Dingding Zhang, Yuanfen Li, Houbin Zhang, Yibing Yin, Fang Lu, Xianjun Zhu, Zhenglin Yang

**Affiliations:** 1 Department of Laboratory Medicine, Chongqing Medical University, Chongqing, China; 2 Sichuan Provincial Key Laboratory for Human Disease Gene Study, Institute of Laboratory Medicine, Hospital of the University of Electronic Science and Technology of China and Sichuan Provincial People’s Hospital, Chengdu, China; 3 Medicine Information Center, School of Medicine, University of Electronic Science and Technology of China, Chengdu, Sichuan, China; 4 Department of Genetics, Aravind Medical Research Foundation, Aravind Eye Hospital, Madurai, Tamil Nadu, India; 5 Retina-Vitreous Services, Aravind Eye Hospital, Madurai, Tamil Nadu, India; 6 Chengdu Institute of Biology, Chinese Academy of Sciences, Chengdu, China; 7 Sichuan Translational Medicine Hospital, Chinese Academy of Sciences, Chengdu, China

## Abstract

Retinitis pigmentosa (RP) is a rare heterogeneous genetic retinal dystrophy disease, and despite years of research, known genetic mutations can explain only approximately 60% of RP cases. We sought to identify the underlying genetic mutations in a cohort of fourteen Indian autosomal recessive retinitis pigmentosa (arRP) families and 100 Indian sporadic RP cases. Whole-exome sequencing (WES) was performed on the probands of the arRP families and sporadic RP patients, and direct Sanger sequencing was used to confirm the causal mutations identified by WES. We found that the mutations of *EYS* are likely pathogenic mutations in two arRP families and eight sporadic patients. Specifically, we found a novel pair of compound heterozygous mutations and a novel homozygous mutation in two separate arRP families, and found two novel heterozygous mutations in two sporadic RP patients, whereas we found six novel homozygous mutations in six sporadic RP patients. Of these, one was a frameshift mutation, two were stop-gain mutations, one was a splicing mutation, and the others were missense mutations. In conclusion, our findings expand the spectrum of *EYS* mutations in RP in the Indian population and provide further support for the role of *EYS* in the pathogenesis and clinical diagnosis of RP.

Retinitis pigmentosa (RP; OMIM 226800) is a highly heterogeneous genetic disease characterized by progressive visual loss caused by the impairment of retinal photoreceptors^1^. The worldwide prevalence of RP is approximately one in 3,500–5,000^2^, and it can be inherited as an autosomal recessive (50–60%), an autosomal dominant (30–40%) or an X-linked trait (5%–15%). Thus far, more than 70 genes and loci have been identified for RP (https://sph.uth.edu/RetNet/). However, these genes account for only approximately 60% of RP cases^3^. Therefore, unknown RP genes remain to be identified, and novel RP genes would provide valuable information for the diagnosis, prevention and treatment of RP.

The *EYS* gene (OMIM 612424, NM_001142800) corresponds to the RP25 locus and was identified as the gene causing autosomal recessive retinitis pigmentosa (arRP) in 2008, and it is mainly expressed in the retina^4^. The human *EYS* gene encodes a homologue of the Drosophila eye spacemaker (SPAM) protein and is essential for the development and morphology of photoreceptors^5^. Thus far, 15 mutations have been reported in *EYS* for RP patients, and the types of mutations include missense mutations, nonsense mutations, insertions, deletions and splice site mutations^2^,^6^,^7^,^8^,^9^,^10^.

However, there has been limited success when using traditional approaches to screen potential genes for RP because many techniques for positional cloning and gene identification are relatively time consuming, expensive and inefficient. Recently, whole-exome sequencing (WES) by next-generation sequencing (NGS) has become an efficient method for identifying genetic variants at the whole-genome level. In several studies, NGS has provided a promising alternative approach for the molecular diagnosis and genetic identification of RP^11^,^12^. Because of relatively high levels of consanguinity and large numbers of offspring per family, hereditary-disease gene analysis is highly effective in the Indian population. In this study, which is part of an international collaborative project, we used WES to identify disease-causing genes for RP in the Indian Population, and the results indicated novel mutations in the *EYS* gene in two consanguineous Indian families and eight sporadic Indian RP patients. Our findings expand the mutation spectrum of *EYS* within the Indian population and demonstrate that WES by NGS is a powerful tool for the genetic diagnosis of RP.

## Methods

### Patient recruitment and ethics statement

All study protocols were approved by the Ethics Review Board of Aravind Medical Research Foundation of India and the Hospital of the University of Electronic Science and Technology of China and the Sichuan Provincial People’s Hospital, and all experiments were performed in accordance with the approved protocols. Fourteen families with arRP, 100 patients with sporadic RP and 1000 control individuals with no history of retinal diseases were recruited in this study. Samples were collected from the Aravind Eye Hospital of India. Written informed consent was obtained from all individuals who participated in this study or from their legal guardians in the case of minors. Venous blood samples were obtained from all subjects in EDTA vacutainers.

### DNA extraction

Total genomic DNA was isolated with DNA extraction kits according to the manufacturer’s instructions (TianGen, Beijing, China) and stored at −20 °C for later use. The integrity of the DNA was assessed through 1% agarose gel electrophoresis.

### Whole-exome sequencing and data analysis

DNA samples from the probands of arRP families and 100 sporadic RP patients were subjected to WES at Axeq Technology Inc., Seoul, Korea. In brief, the workflow of WES was as follows. First, the genomic DNA samples were fragmented into 150–200 bp fragments and then ligated to paired-end adaptors. Exome enrichment was performed according to the manufacturer’s protocol with the Agilent SureSelect Human All Exon 50 MB kit V5 (Santa Clara, Californian, U.S.A.), which covers 20,965 genes and 334,378 exons in the Consensus Coding Sequence Region database. The captured libraries were subjected to a quality assessment with an Axeq 2100 Bioanalyzer and sequenced on an Illumina HiSeq 2000 sequencer. The raw image files were processed by Illumina base calling software v.1.7 for base calling with default parameters, and the sequences of each individual were generated as 90-bp paired-end reads. High-quality sequencing reads of each sample were aligned to the reference human genome hg19 UCSC assembly (http://genome.ucsc.edu/) using the Burrows-Wheeler Aligner (BWA) program v.0.5.9-r16 (http://bio-bwa.sourceforge.net/)^13^. The SNPs and indels were detected by SAMTOOLS v.0.1.19 (http://samtools.sourceforge.net/) using the ‘mpileup’ command. The variants that had depths less than 10 and those located outside of the exome-capture regions were filtered out. For all of the samples, we used individual base-calling algorithms.

SnpEff v4.1 software was used for variants annotation (http://sourceforge.net/projects/snpeff/files/databases/v4_1/).

### Gene filtration and annotation

The detected variants were filtered and annotated based on four databases: NCBI CCDS (http://www.ncbi.nlm.nih.gov/CCDS/ccdsBrowse.cgi), RefSeq (http://www.ncbi.nlm.nih.gov/RefSeq/), Ensembl (http://www.ensembl.org), and Encode (http://genome.ucsc.edu/ENCODE). Exclusion steps were performed to identify the candidate variants. Variants within intergenic, intronic and UTR regions and synonymous mutations were excluded, and variants in dbSNP138 (http://www.ncbi.nlm.nih.gov/projects/SNP/), the 1000 Genome project (ftp://ftp.1000 genomes.ebi.ac.uk/vol1/ftp), the YH database (http://yh.genomics.org.cn/), the HapMap Project (ftp://ftp.ncbi.nlm.nih.gov/hapmap) and our in-house database generated from 1600 samples sequenced by WES were also excluded. Moreover, potentially damaging effects of the variants on protein structure/function were predicted by SIFT (http://sift.bii.astar.edu.sg/) and Ployphen2 (http://genetics.bwh.havard.edu/pph2).

### Sanger sequencing

Sanger sequencing was performed to confirm the variants identified by WES. Primers flanking the candidate loci were designed using Primer 5.0 and synthesized by Invitrogen, Shanghai, China. The primers used for Sanger sequencing are shown in supplementary Table S1. PCR amplification was performed, and the products were purified using the PCR purification kit from Qiagen following the manufacturer’s instructions. The purified PCR products were then sequenced on an ABI3730 genetic analyser. The sequencing results were compared with the *EYS* gene reference sequence (NC_000006.12) to confirm the candidate nucleotide variants.

## Results

### Clinical features of Indian families with arRP and sporadic RP

**Figure 1:**
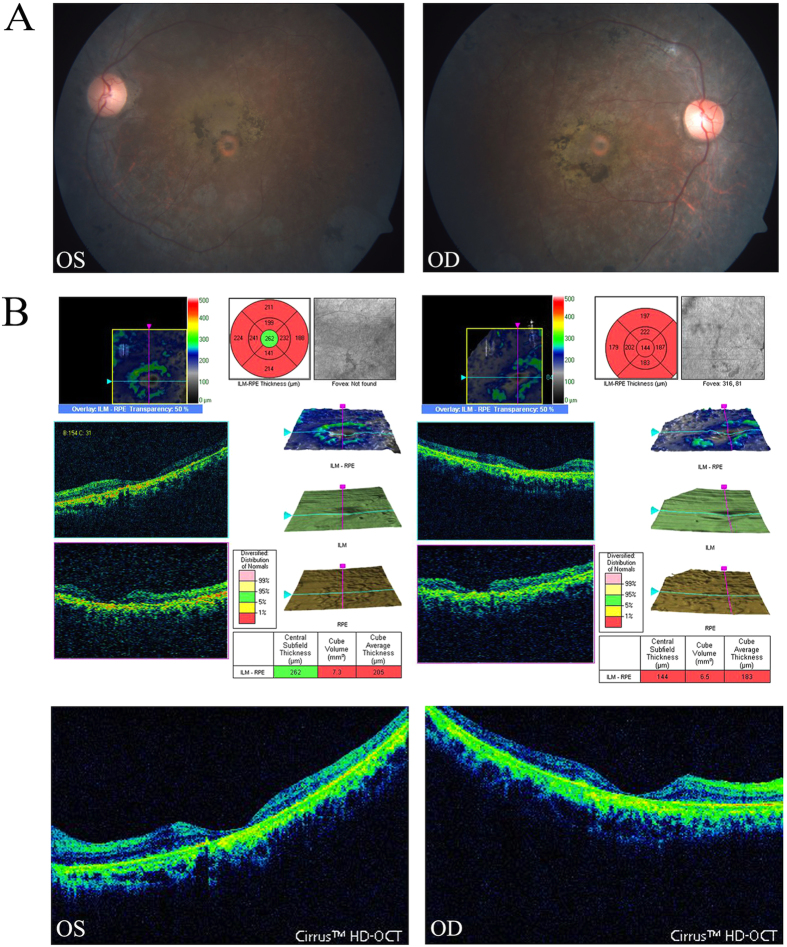
Fundus photographs and OCT pictures of the proband in family ARRP-49. The fundus photographs (**A**) show typical changes (waxy-pale disc, arteriolar attenuation and bone-spicule pigment) of fundus in the left eye (OS LE) and right eye (OD RE); the OCT pictures of the left and right eyes (**B**) show atrophy of the foveal and retinal layers (macular thickness: macular cube 512 × 128).

**Figure 2:**
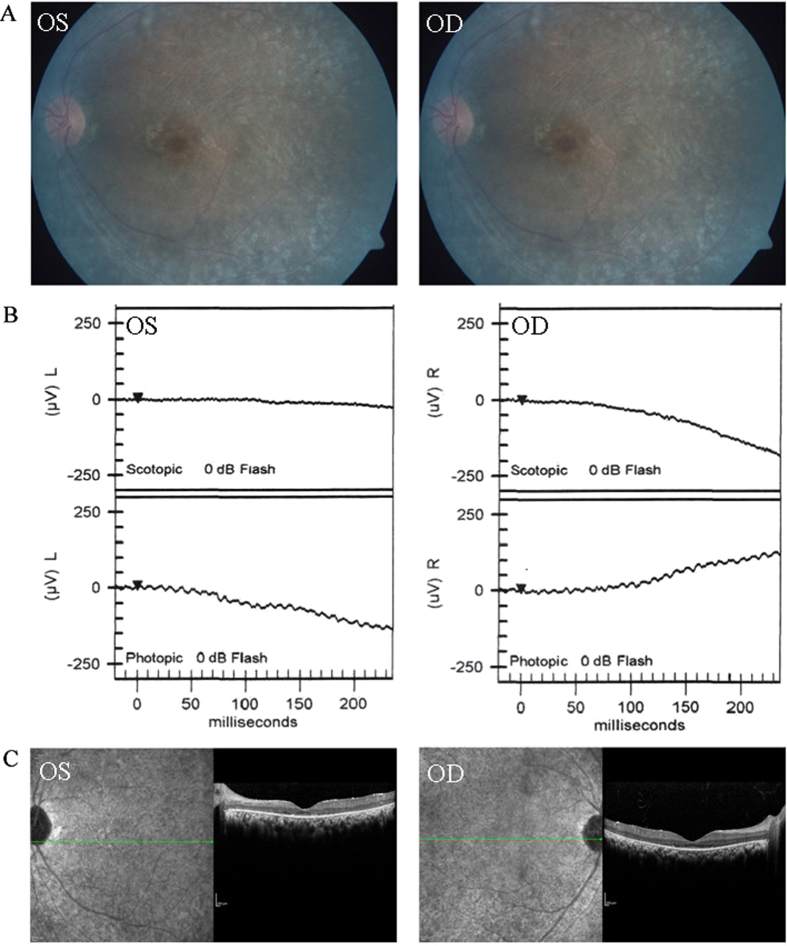
Representative photographs of sporadic patients RP:S-14. Fundus photographs (**A**) showed bone-spicule pigment and arteriolar attenuation; flash ERG (**B**) showed a-wave or b-wave with reduced or extinguished amplitude; OCT picture (**C**) showed thinner and atrophy retina.

**Figure 3:**
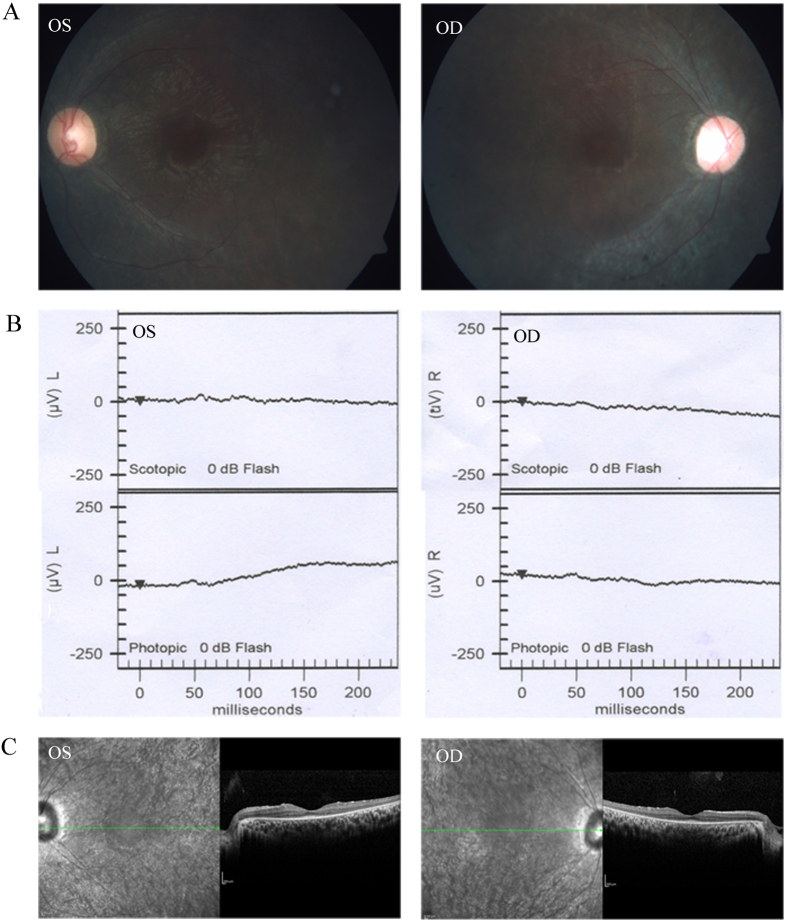
Representative photographs of sporadic patients RP:S-48. Fundus photographs (**A**) showed bone-spicule pigment and arteriolar attenuation; flash ERG (**B**) showed a-wave or b-wave with reduced or extinguished amplitude; OCT picture (**C**) showed thinner and atrophy retina.

**Figure 4:**
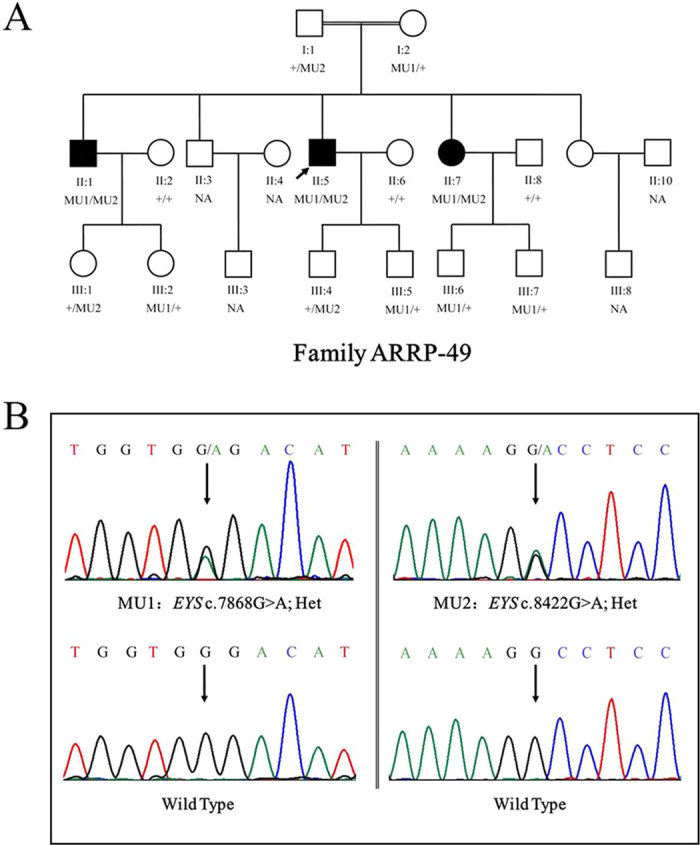
Pedigree of the family ARRP-49 and mutations identified by Sanger sequencing analysis. A shows the segregation of compound heterozygous changes c.8422G>A (MU1) and c.7868G>A (MU2). Genotypes are presented as follows: MU1/MU2 represents individuals with both mutations as compound heterozygous; MU1/+ and +/MU2 indicate heterozygous carriers; +/+ indicates individuals carrying two wild-type alleles. NA denotes DNA samples that were unavailable.

**Figure 5:**
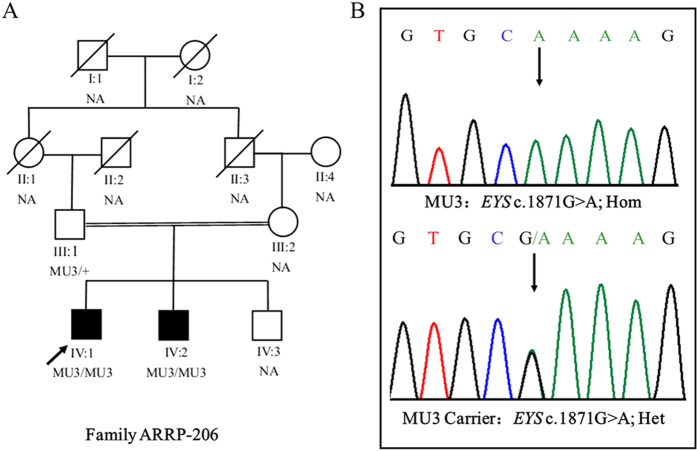
Pedigree of the family ARRP-206 and mutations identified by Sanger sequencing. A shows the segregation of a homozygous mutation c.1871G>A (MU3). The proband is indicated by an arrow. MU3/MU3 represents a homozygous mutant, whereas MU3/+ indicates a heterozygous carrier. Black symbols indicate affected individuals; white symbols indicate unaffected individuals. NA denotes DNA samples that were unavailable.

The detailed clinical data for two arRP families and eight sporadic RP patients are shown in Table 1. Ophthalmic examinations identified two affected members in family ARRP-206 and three affected members in ARRP-49. The sporadic patients and patients in the arRP families exhibited similar clinical features of RP, such as night blindness and low visual acuity. Representative fundus photographs and optical coherence tomography (OCT) are shown in Fig. 1 (ARRP-49), Fig. 2 (SP-14) and Fig. 3 (SP-48), which indicates an obvious waxen appearance of the discs, bone-spicule pigmentation in the midperiphery, attenuation of the retinal arteries (Fig. 1A) and atrophy of the foveal and retinal layer (Fig. 1B). The patients’ parents and other unaffected family members did not show any RP features. ARRP-49 and ARRP-206 showed an autosomal recessive inherited pattern (Figs 4A and 5A).

### Mutation analysis using whole-exome and Sanger sequencing

WES was conducted on the probands of 14 arRP families and 100 sporadic RP patients. The mean read depth of the target regions of each sample ranged from 52-66X, and the average throughput depth of the target region in each sample ranged form 84-104.9X (See supplementary table S2 for detailed information.) To identify the underlying genetic mutations, we focused on the functional SNPs/indels with a homozygous or compound heterozygous status, including nonsynonymous variants, splice acceptor and donor site mutations, and frameshift insertions or deletions. These variants were compared with 5 SNP databases (dbSNP138, the 1000 Genomes Project, the HapMap Project, the YH database and the in-house database). Finally, three compound heterozygous mutations and seven homozygous mutations in the *EYS* gene were identified in two arRP families and eight sporadic RP patients. The detailed WES results for these patients are illustrated in supplementary table S2, and NGS results for the SNP quality and depth of these mutations are listed in Table S3. In family ARRP-49, we used the recessive compound heterozygous inheritance model and found one pair of compound heterozygous mutations (c.8422G>A (p.A2808T) and c.7868G>A (p.G2623E)) (Table 2) in the *EYS* gene (NM_001142800). In family ARRP-206, we found a homozygous single-nucleotide mutation (c.1871G>A (p.S624L)) (Table 2) in ARRP-206. To confirm the accuracy of the mutations identified by exome sequencing, PCR-based Sanger sequencing was performed to validate these mutations in the other family members, and the results demonstrated that the parents were unaffected carriers of c.7868G>A (p.G2623E, father) and c.8422G>A (p.A2808T, mother) in family ARRP-49 and c.1871G>A (p.S624L, father) in family ARRP-206. The inheritance pattern showed complete co-segregation of the mutations with the disease phenotype (Figs 2 and 3). The detailed genotype results of the family members in ARRP-49 and ARRP-206 are illustrated in supplementary Table S4.

**Figure 6:**
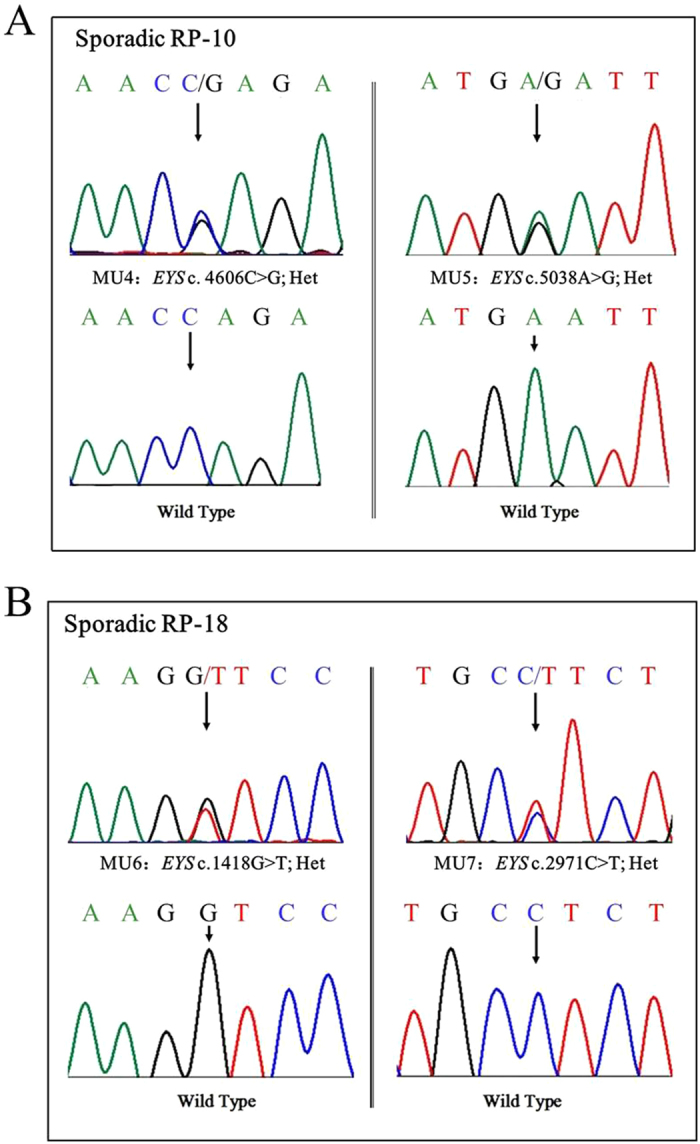
Sanger sequencing in sporadic patients RP-S:10 and RP-S:18 (**A** and **B**) showed 2 compound heterozygous changes.

**Figure 7:**
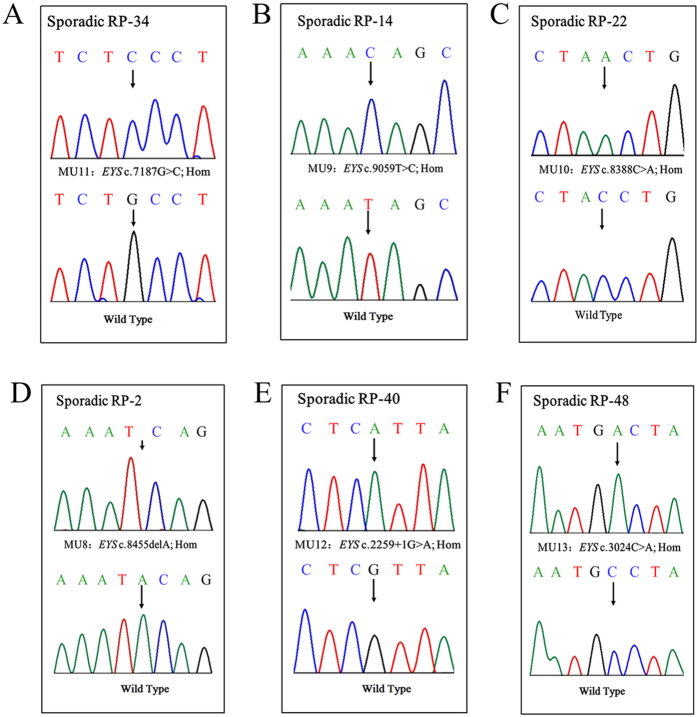
Sanger sequencing in sporadic patients RP-S:2, RP-S:22, RP-S:14, RP-S:34 and RP-S:48 showed homozygous mutations.

In 100 sporadic RP patients, we found two compound heterozygous mutations in two patients, RP:S-10 and RP:S-18 (Table 2, Fig. 6A,B), and six novel likely pathogenic mutations in six patients (Table 2, Fig. 7). These mutations included two stop-gain mutations, one splicing mutation, six missense mutations, and one frameshift deletion (Table 2). These mutations either affect predicted functional regions of *EYS*, such as the Laminin G (LamG), EGF, EGF-like calcium-binding (EGF-CA), and EGF-like domains (Fig. 6A), or affect evolutionarily conserved amino acid residues (Fig. 6B).

Additional disease-causing mutations of other known RP genes or previously identified mutations of *EYS* were not found in any of these patients. According to the Exome Aggregation Consortium database (http://exac.broadinstitute.org/) and our 1000 control results, homozygous mutations or compound heterozygous mutations were not detected in the normal control population.

### Mutation prediction analysis of the *EYS* gene

**Figure 8:**
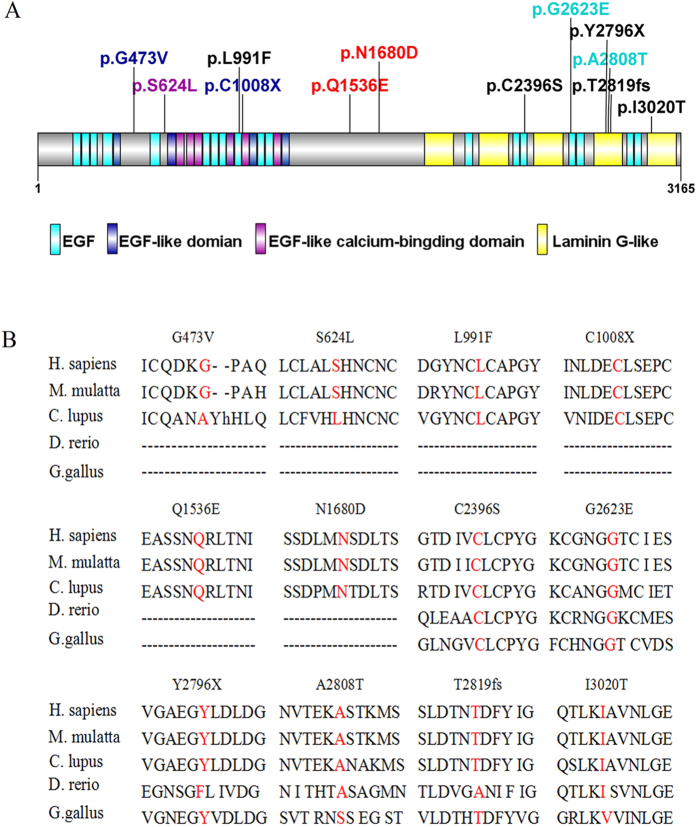
Distribution of *EYS* mutations identified in this study and sequence alignment of the affected amino acid residues. (**A**) Distribution of mutations on the various domains of the *EYS* protein. (**B**) Amino acid sequence comparison of *EYS* among homologous genes in Euteleostomi.

SIFT, PROVEAN^14^ and SMART were used to predict the effect of the identified amino acid substitutions on the *EYS* protein function. Most mutations are located at the protein functional domain LamG or EGF/EGF-like/EGF-CA, which may affect the function of the protein (Table 2, Fig. 8A).

## Discussion

*EYS* spans 2.0 Mb of genomic DNA and encodes 3,165 amino acids, and it is considered one of the largest genes expressed in the human eye. *EYS* is a multi-domain protein that starts with a signal peptide of 21 amino acids. It contains 28 epidermal growth factor (EGF) or EGF-like domains at the N-terminus and five LamG domains at the C-terminus and is highly expressed in retinal photoreceptors^9^. Previous studies have shown that a homologous protein of *EYS* in *Drosophila* named Spacemaker (spam) is involved in luminal space formation and plays an essential role in the formation of matrix-filled interrhabdomeral space in *Drosophila*^15^,^16^,^17^. However, its biological function in the human retina remains unknown. Further investigations for *EYS* function in the retina are essential for deep understanding of the pathological mechanism of retinal degeneration.

*EYS* is a major causative gene for RP. Thus far, the mutations identified in *EYS* include p.D904Qfs*17, p.S754Afs*6, p.T657Afs*5, p.W2640*, p.E1836* and others^5^,^9^,^18^. Most of these reported mutations result in a truncated *EYS* protein, which reveals that the C-terminus of *EYS* is essential for its function in the retina. In this study, we found a pair of compound heterozygous mutations, c.8422G>A (p.A2808T) and c.7868G>A (p.G2623E), in *EYS* in family ARRP-49. Both mutations affect a conserved amino acid residue. The mutation p.A2808T in exon 43 is a single nucleotide polymorphism (rs111991705) with an allele frequency of 0.009826. This mutation results in the replacement of a small hydrophobic amino acid (alanine) by a polar residue (threonine). Previously, Audo, I., *et al.* reported the same heterozygous mutation c.8422G>T in a French RP patient^18^. This French RP patient had another heterozygous mutation c.3329C>G^18^. The novel mutation p.G2623E in exon 40 identified in our study lies in a region of disulphide bond modification (http://www.uniprot.org/uniprot/Q5T1H1), which may affect the formation of disulphide bonds, thereby impairing protein function. These two mutations together may account for the incidence of RP in family ARRP-49. The novel homozygous mutation c.1871G>A (p.S624L) in *EYS* is likely the causative mutation for family ARRP-206. This missense mutation leads to the replacement of a small-sized polar residue, serine, by a hydrophobic lysine. However, this mutation is not highly evolutionarily conserved, and further studies are warranted to ascertain the impact of this mutation on *EYS* functions.

The majority of RP cases show the sporadic form^19^,^20^, and the inheritance pattern of this form is difficult to ascertain. In our study, we found two compound heterozygous mutations in the sporadic RP patients RP:S-10 and RP:S-18. In patient RP:S-10, the compound heterozygous mutations c.C4060G (p.Q1536E) and c.A5038G (p.N1680D) were found, and these mutations are located in exon 26 and the unknown region of the protein, respectively. In patient RP:S-18, the compound heterozygous mutations c.G1418T (p.G473V) and c.C2971T (p.L991F) were found, and these mutations are located in the unknown region and the EGF domain of the protein, respectively. However, there were several limitations in this study. Because the blood samples of the intra-family members of the sporadic RP patients were difficult to collect, we could not assess the genotypes of these intra-family members, which included their parents, siblings or offspring. Therefore, we could not be certain whether these two variants originate from distinct alleles or the same allele in these two sporadic patients.

In sporadic patients RP:S-22 and RP:S-48, we found two novel homozygous stop-gain mutations, c.8388C>A (p.Y2796X) and c.3024C>A (p.C1008X), respectively, and these mutations resulted in the generation of truncated proteins in the fourth LamG domain at the C-terminal and the fifth EGF-CA domain at the N-terminal, respectively. The truncated proteins likely impair the function of *EYS*. These nonsense mutations could cause the degradation of *EYS* mRNA via nonsense-mediated mRNA decay (NMD)^21^. We also found a novel frameshift deletion mutation in exon 43 and a splicing-site mutation in exon 15 in patients RP:S-2 and RP:S-40. Both mutations led to abnormal proteins. In sporadic patients RP:S-14 and RP:S-34, we found missense mutations in the LamG and EGF domains, respectively, and the SIFT/PROVEAN predictions indicate that these mutations may be harmful to the protein. Iwanami *et al.*, demonstrated that mutation types were related to the severity of the RP symptoms^22^. In our study, the correlation between genotype and phenotype of patients is not readily obvious due to the small number of subjects. More studies about the correlation between genotype and phenotype of RP would give the better understanding of the diagnosis and prediction of the incidence of RP.

In summary, we identified three novel compound heterozygous mutations in *EYS* and seven novel homozygous mutations for RP in the Indian population. Our study not only expands the spectrum of *EYS* mutations for arRP in the Indian population but also shows that WES can be an effective tool for identifying causative mutations in RP patients and diagnosing genetic diseases.

## Additional Information

**How to cite this article**: Di, Y. *et al.* Whole-exome Sequencing Analysis Identifies Mutations in the *EYS* Gene in Retinitis Pigmentosa in the Indian Population. *Sci. Rep.* **6**, 19432; doi: 10.1038/srep19432 (2016).

## Supplementary Material

Supplementary Information

## Figures and Tables

**Table 1:** Clinical features of patients with RP.

Patients ID	Mutations	Age (year)/Sex	Onset Age (year)	ERG		Fundus Appearance	
				LE	RE	LE	RE
ARRP-49	c.[8422G>A];[7868G>A]	50/M	32	NA	NA	PD/ODW/RR	PD/ODW/RR
ARRP-206	c.[1872g>A]	21/M	15	NA	NA	NA	NA
RP:S-2	c.[8455delA]	8/M	1	NA	NA	NA	NA
RP:S-10	c.[4606C>G];[5038A>G]	55/M	40	NA	NA	NA	NA
RP:S-14	c.[9059T>C]	14/M	0	R-a/b	R-a/b	PD/ODW	PD/ODW
RP:S-18	c.[1418G>T];[2971C>T]	55/M	52	NA	NA	NA	NA
RP:S-22	c.[8388C>A]	38/M	13	NA	NA	NA	NA
RP:S-34	c.[7187G>A]	44/F	14	NA	NA	NA	NA
RP:S-40	c.[2259+1G>A]	25/M	13	NA	NA	NA	NA
RP:S-48	c.[3024C>A]	20/F	15	R-a/b	R-a/b	PD/ODW	PD/ODW

Abbreviations: M: male; F: female; LE, left eye; RE, right eye; ERG: electroretinography; R-a/b: a or/and b-wave with reduced amplitude; ODW: optic disk waxy; AA, artery attenuation; PD: pigment deposits; NA: not available.

**Table 2:** Mutations identified in the present study.

Family ID	Exon	Nucleotide Mutations	Allele State	Protein effect	Mutation type	dbSNP ID	Protein locus	SIFT/PROVEN(Cutoff0.05/-2.5)
ARRP-49	43	c.8422G>A	het	p.A2808T	missense	rs111991705	LamG	Tolerated (0.051)/Neutral (–0.61)
ARRP-49	40	c.7868G>A	het	p.G2623E	missense	novel	EGF	Damaging (0.009)/Deleterious (–2.84)
ARRP-206	12	c.1871G>A	hom	p.S624L	missense	novel	Unknown region	Tolerated (0.006)/Neutral (1.69)
RP:S-2	43	c.8455delA	hom	p.T2819fs	frameshift_del	novel	LamG	NA/NA
RP:S-10	26	c.4606C>G	het	p.Q1536E	missense	novel	Unknown region	Damaging (0.000)/Neutral (0.388)
RP:S-10	26	c.5038A>G	het	p.N1680D	missense	novel	Unknown region	Damaging (0.000)/Neutral (-0.63)
RP:S-14	43	c.9059T>C	hom	P.I3020T	missense	novel	LamG	Damaging (0.000)/Neutral (–0.65)
RP:S-18	9	c.1418G>T	het	p.G473V	missense	novel	Unknown region	NA/NA
RP:S-18	19	c.2971C>T	het	p.L991F	missense	novel	EGF	Tolerated (0.541)/Neutral (–1.53)
RP:S-22	43	c.8388C>A	hom	p.Y2796X	stopgain	novel	LamG	NA/NA
RP:S-34	36	c.7187G>C	hom	p.C2396S	missense	novel	EGF	Damaging (0.000)/Deleterious (-3.84)
RP:S-40	15	c.2259+1 G>A	hom	–	splicing	novel	–	NA/NA
RP:S-48	20	c.3024C>A	hom	p.C1008X	stopgain	novel	EGF-CA	NA/NA
