# Supplementary material for: Whole-exome Sequencing Analysis Identifies Mutations in the EYS Gene in Retinitis Pigmentosa in the Indian Population
Source: Sci Rep. 2016 Jan 20;6:19432. doi: 10.1038/srep19432 (PMC4726297; doi:10.1038/srep19432)
Supplement: Supplementary Information [file srep19432-s1.doc]

Whole-exome Sequencing Analysis Identifies Mutations in the *EYS* Gene in Retinitis Pigmentosa in the Indian Population

Yanan Di^1,2,3,7^*, Lulin Huang^2,3,7^*, Periasamy Sundaresan^4^*, Shujin Li^2,6,7^, Ramasamy Kim^5^, Bibhuti Ballav Saikia^4^, Chao Qu^2,7^, Xiong Zhu^2,3,7^, Yu Zhou^2,3,7^, Zhilin Jiang^2,7^, Lin Zhang^2,6,7^, Ying Lin^2,7^, Dingding Zhang^2,7^, Yuanfen Li^2,7^, Houbin Zhang^2,3,7^, Yibing Yin^1^, Fang Lu^2,3,7^, Xianjun Zhu^2,3,6,7#^, Zhenglin Yang^1,2,3,6,7#^

^*^These authors contributed equally to this study.

**Supplementary information file**

Supplementary information file includes three Tables (**Table S1, S2，S3 and S4**).

Table S1 The primer pairs used for mutations identification by Sanger Sequence

| Mutations | Primer name |  | Primer pairs 5’-3’ |
| --- | --- | --- | --- |
| c.8422G>A | *EYS*-8422 | F | CTGGCAAACATCTGCAAGAA |
|  |  | R | ATCCAACTTGGCCAGAAACA |
| c.7868G>A | *EYS*-7868 | F | ATGGCATAAATGCTGTGCTG |
|  |  | R | TTCTCTGCGCATTTCTGTATTC |
| c.4606C>G | *EYS-*4606,5038 | F | GCCTCCATAAGTGCAACTCC |
|  |  | R | CACTTGGGTGAAGTTTGAACAG |
| c.5038A>G | *EYS-*4606,5038 | F | GCCTCCATAAGTGCAACTCC |
|  |  | R | CACTTGGGTGAAGTTTGAACAG |
| c.9059T>C | *EYS-9059* | F | TGCAGAAATGGAGGTGAATG |
|  |  | R | CCATATTCAAAGCCCCCTAGA |
| c.1418G>T | *EYS*-1418 | F | TCACTGTGGTTTTAAAAATTAGCTG |
|  |  | R | CCATTAACCACTCCCTTCCA |
| c.2971C>T | *EYS*-2971 | F | TGGTTTCCAGCTTCATCCAT |
|  |  | R | ATTTTTGCCCTGTTTGCATC |
| c.8455delA | *EYS*-8455 | F | TCCGTTCAACTTCGCTACAA |
|  |  | R | TCACCTCCATTTCTGCATGT |
| c.8388C>A | *EYS*-8388 | F | TCCGTTCAACTTCGCTACAA |
|  |  | R | TCACCTCCATTTCTGCATGT |
| c.7187C>G | *EYS*-7187 | F | GCATATGTGTTCATGCATGTGT |
|  |  | R | CCTGCTTGGTGATCAGTCTC |
| c.2259+1 G>A | *EYS*-splicing | F | TGTTTTTTCCAGTGGTTGATG |
|  |  | R | AAACCAACCTGTATAGAGTGGAGA |
| c.3024C>A | *EYS*-3024 | F | GAGGGTCTTCATTTCTTGGTGATG |
|  |  | R | TCAACTTTCCCTTGATGTTAAGTC |

**Table S2 Overview of data production**

| **Items** | **ARRP 49-II:5** | **ARRP 206-IV:1** | **RP:S-2** | **RP:S-10** | **RP:S-14** |
| --- | --- | --- | --- | --- | --- |
| *Total reads* | 49,8119,518 | 48,389,056 | 43,538,982 | 44,372,220 | 48,018,436 |
| *Total yield (bp)* | 5,031,771,318 | 4,887,294,656 | 4,397,437,182 | 4,481,594,220 | 4,849,862,036 |
| *Average throughput depth of target region* | 99.9 | 97.0 | 87.3 | 88.9 | 96.2 |
| *Mapped to human genome* | 49,563,492 | 48,211,940 | 43,388,304 | 44,242,436 | 47,877,526 |
| *De-duplicated by Picard tools* | 48,277,967 | 47,041,876 | 41,323,899 | 42,827,662 | 45,442,035 |
| *Uniquely mapped to human genome* | 46,960,891 | 45,862,952 | 40,264,663 | 41,770,934 | 44,292,449 |
| *Mapped to target regions* | 35,099,450 | 35,466,449 | 33,097,880 | 33,955,987 | 35,627,912 |
| *% Coverage of target regions ≥1X* | 98.1% | 98.2% | 97.6% | 98.0% | 98.2% |
| *% Coverage of target regions ≥10 X* | 94% | 94.9% | 91.4% | 93.2% | 94,2% |
| *Mean read depth of target regions* | 58.3 | 58.7 | 55.5 | 56.3 | 58.8 |
| *Number of SNPs* | 66,992 | 67,419 | 60,462 | 63,932 | 65,656 |
| *Number of coding SNPs* | 20,370 | 20,510 | 19,310 | 19,869 | 20,126 |
| *Number of synonymous SNPs* | 10,557 | 10,703 | 10,027 | 10,299 | 10,525 |
| *Number of nonsynonymous SNPs* | 9,336 | 9,325 | 8,805 | 9,102 | 9,096 |
| *Number of Indels* | 5,541 | 5,621 | 4,526 | 5.001 | 5,151 |
| *Number of coding Indels* | 413 | 427 | 381 | 411 | 397 |

**Table S2 Overview of data production (continued)**

| **Items** | **RP:S-18** | **RP:S-22** | **RP:S-34** | **RP:S-40** | **RP:S-48** |
| --- | --- | --- | --- | --- | --- |
| *Total reads* | 52,316,262 | 49,518,246 | 46,810,310 | 41,988,552 | 48,627,488 |
| *Total yield (bp)* | 5,283,942,462 | 5,001,342,846 | 4,727,841,310 | 4,240,843,752 | 4,911,376,288 |
| *Average throughput depth of target region* | 104.9 | 99.3 | 93.8 | 84.2 | 97.5 |
| *Mapped to human genome* | 52,138,722 | 49,363,126 | 46,629,684 | 41,854,296 | 48,474,470 |
| *De-duplicated by Picard tools* | 49,538,198 | 47,303,076 | 44,118,412 | 40,213,737 | 46,857,500 |
| *Uniquely mapped to human genome* | 48,283,596 | 46,128,933 | 42,999,222 | 39,173,334 | 45,702,299 |
| *Mapped to target regions* | 39,579,744 | 37,029,371 | 35,063,689 | 31,423,908 | 35,869,218 |
| *% Coverage of target regions ≥1X* | 97.9% | 98.0% | 97.7% | 98.0% | 98.0% |
| *% Coverage of target regions ≥10 X* | 93.2% | 93.4% | 91.8% | 92.6% | 93.9% |
| *Mean read depth of target regions* | 66.2 | 61,1 | 58.7 | 52.4 | 59.5 |
| *Number of SNPs* | 64,689 | 64,050 | 63,228 | 62,018 | 64,465 |
| *Number of coding SNPs* | 20,273 | 19,841 | 19,843 | 19,218 | 19,624 |
| *Number of synonymous SNPs* | 10,552 | 10,291 | 10,419 | 9,964 | 10,244 |
| *Number of nonsynonymous SNPs* | 9,248 | 9,045 | 8,951 | 8,740 | 8,935 |
| *Number of Indels* | 4,898 | 4,906 | 4,526 | 4,945 | 5,193 |
| *Number of coding Indels* | 4 | 413 | 389 | 414 | 440 |

**Table S3. The SNP quality and depth by NGS of each identified mutation**

| Patient ID | Mutations | SNP Quality | Total depth | Alternate depth |
| --- | --- | --- | --- | --- |
| ARRP-49 | c.8422G>A | 179 | 45 | 24 |
| ARRP-49 | c.G7868A | 97 | 56 | 21 |
| ARRP-206 | c.1871G>A | 157 | 101 | 101 |
| RP:S-2 | c.8455delA | 214 | 99 | 99 |
| RP:S-10 | c.4606C>G | 124 | 78 | 38 |
| RP:S-10 | c.5038A>G | 194 | 101 | 50 |
| RP:S-14 | c.9059T>C | 173 | 106 | 106 |
| RP:S-18 | c.1418G>T | 201 | 86 | 43 |
| RP:S-18 | c.2971C>T | 167 | 57 | 28 |
| RP:S-22 | c.8388C>A | 191 | 138 | 136 |
| RP:S-34 | c. 7187 G>C | 196 | 62 | 62 |
| RP:S-40 | c.2259+1 G>A | 222 | 75 | 75 |
| RP:S-48 | c. 3024 C>A | 222 | 98 | 98 |

**Table S4 The genotype of family ARRP-49** **members**

| Family member | Sex | Mutations |
| --- | --- | --- |
| I:1 | M | +/MU2 |
| I:2 | F | MU1/+ |
| II:1 | M | MU1/MU2 |
| II:2 | F | +/+ |
| II:5 | M | MU1/MU2 |
| II:6 | F | +/+ |
| II:7 | F | MU1/MU2 |
| II:8 | M | +/+ |
| III:1 | F | +/MU2 |
| III:2 | F | MU1/+ |
| III:4 | M | +/MU2 |
| III:5 | M | MU1/+ |
| III:6 | M | MU1/+ |
| III:7 | M | MU1/+ |

*M: male; F: female；MU1：c.7868G>A, p.2623G>E; MU2: c.8422G>A, p.2808A>T; MU3: c.1871G>A, p.624S>L.
